# ABO Blood-Typing Using an Antibody Array Technique Based on Surface Plasmon Resonance Imaging

**DOI:** 10.3390/s130911913

**Published:** 2013-09-09

**Authors:** Nongluck Houngkamhang, Apirom Vongsakulyanon, Patjaree Peungthum, Krisda Sudprasert, Pimpun Kitpoka, Mongkol Kunakorn, Boonsong Sutapun, Ratthasart Amarit, Armote Somboonkaew, Toemsak Srikhirin

**Affiliations:** 1 Materials Science and Engineering Programme, Faculty of Science, Mahidol University, Rama 6 Rd., Phayathai, Rajathavee, Bangkok 10400, Thailand; E-Mails: luck2527@gmail.com (N.H.); por_jubjub@hotmail.com (P.P.); srati_wonnuch@hotmail.com (K.S.); 2 Department of Pathology, Faculty of Medicine, Ramathibodi Hospital, Mahidol University Rama 6 Rd., Phayathai, Rajathavee, Bangkok 10400, Thailand; E-Mails: apirom_odd@yahoo.com (A.V.); pimpun.tar@mahidol.ac.th (P.K.); mongkol.kun@mahidol.ac.th (M.K.); 3 School of Electronic Engineering and School of Telecommunications Engineering, Suranaree University of Technology, 111 University Avenue, Muang District, Nakhon Ratchasima 30000, Thailand; E-Mail: boonsong@sut.ac.th; 4 Photonics Technology Laboratory, National Electronics and Computer Technology Center (NECTEC), Pathumthani 12120, Thailand; E-Mails: ratthasart.amarit@nectec.or.th (R.A.); armote.somboonkaew@nectec.or.th (A.S.); 5 Physics Department, Faculty of Science, Mahidol University, Rama 6 Rd., Phayathai, Rajathavee, Bangkok 10400, Thailand

**Keywords:** ABO blood typing, surface plasmon resonance imaging (SPR imaging), immunoassay, antibody arrays

## Abstract

In this study, readily available antibodies that are used in standard agglutination tests were evaluated for their use in ABO blood typing by a surface plasmon resonance imaging (SPR imaging) technique. Five groups of antibodies, including mixed clones of anti-A, anti-B, and anti-AB, and single clones of anti-A and anti-B, were used to construct the five-line detection arrays using a multichannel flow cell in the SPR imager. The red blood cell (RBC) samples were applied to a multichannel flow cell that was orthogonal to the detection line arrays for blood group typing. We found that the blood samples were correctly grouped in less than 12 min by the SPR imaging technique, and the results were consistent with those of the standard agglutination technique for all 60 samples. We found that mixed clones of antibodies provided 33%–68% greater change in the SPR signal than the single-clone antibodies. Applying the SPR imaging technique using readily available antibodies may reduce the costs of the antibodies, shorten the measurement time, and increase the throughput.

## 1. Introduction

Surface plasmon resonance (SPR) is an optical technique that measures the surface mass coverage of an adsorbed material [1–4]. SPR is a sensitive technique for studying the interactions between immobilized biomolecules and a solution-phase analyte. The sensitivity and versatility of this label-free, real-time method has advantages for some applications over conventional methods, such as fluorescence or ELISA (enzyme-linked immunosorbent assay). Commercially available SPR systems, such as the Biacore system (Uppsala, Sweden), are well-known and commonly used in many experiments [5–10]. The ABO blood system was the first blood type system [11], and it is also the most clinically important system with regard to blood transfusion. There are several techniques available for blood typing, including gel agglutination [12,13] and SPR [14,15]. The use of SPR to specifically detect the A or B antigens on whole red blood cells (RBCs) was first reported by Quinn *et al.* [16]. The authors reported the high specificity of monoclonal anti-A IgM for A erythrocytes without non-specific binding to B or O erythrocytes. Likewise, a monoclonal anti-B IgM exhibited specific binding to B erythrocytes and no non-specific binding with A or O erythrocytes. Blood from a single donor was used in the experiment for each blood type to avoid variations in the signal between different donors. SPR imaging is a promising platform for use as a high-throughput bioanalyzer in protein analysis [17–19]. These previous reports suggest that there is a possibility of using SPR imaging as a high-throughput technique for ABO blood-typing.

In this work, we evaluated the use of the readily available monoclonal antibodies used in the agglutination technique instead of the purified monoclonal antibody used in previous reports [16] and propose the use of an SPR imager as a detection technique for increasing the throughput. We predict that the use of mixed clones of antibodies may provide coverage for all populations and reduce the cost of the antibodies. In this study, an antibody array of both mixed clones and single clones of anti-A and anti-B was employed to type the ABO blood group in a single run. The results obtained by SPR imaging were compared with those obtained from the conventional agglutination test. The results suggest that the use of the mixed clones of antibodies is preferred over the single clones for ABO blood-typing when using the SPR imaging technique.

## 2. Experimental Section

### 2.1. Reagents

Two types of monoclonal antibodies were used. First, we used mixed clones of monoclonal anti-A, anti-B, and anti-AB (total protein content: 284, 382, and 321 mg/dL, respectively). Additionally, we used single clones of monoclonal anti-A, labeled as 3C4, and anti-B, labeled as 18F8, (total protein content: 324 and 279 mg/dL, respectively). The antibodies and Alsever solution were obtained from the research unit of the Thai Red Cross Society. All antibodies were IgM murine monoclonal antibodies. The blood samples were obtained from the blood bank at Ramathibodi Hospital (Bangkok, Thailand). This work was approved by the Ramathibodi Hospital Ethics Committee. The dextran surfaces (MW 500 kDa) and amine coupling agents (*N*-ethyl-*N*-(3-diethyl-aminopropyl) carbodiimide (EDC), N-hydroxysuccinimide (NHS), ethanolamine, and 0.005% PBST buffer, pH 7.4, were prepared according to the previous work [20].

### 2.2. Sample Preparation

Fresh EDTA blood samples were collected and centrifuged. The plasma was removed, and the RBCs were washed three times with PBS buffer. Alsever preservative was added, and the samples were kept at 4 °C before use. The total number of RBC samples was sixty (n = 15 for each blood group A, B, AB, and O). Suspensions containing 5% RBCs (v/v) were prepared by 1:20 dilution of 100% RBCs in PBS pH 7.4. All procedures followed the AABB guidelines [21].

### 2.3. Agglutination Typing (Test Tube Technique)

A suspension of 3% RBCs was added to a solution of the standard antibodies (mixed-clone antibodies) at 1:2 by volume, centrifuged, and read for agglutination. All of the procedures and interpretation of the results followed the AABB guidelines [21].

### 2.4. SPR Apparatus

The SPR imager and the multichannel flow cell used in this work were developed by the National Electronics and Computer Technology Center (NECTEC), Bangkok, Thailand. SPR imaging, an expansion of conventional SPR, uses a collimated monochromatic light beam with a diameter that is larger than the sensing area. Light reflected off the gold-coated slide is focused onto a CCD camera, allowing for spatial detection of the biomolecular binding events. SPR imaging apparatuses are used at the fixed incidence angle such that the reflected intensity increases linearly with the refractive index change caused by the amount of materials bound to the sensor surface [3]. The details of the SPR imaging setup are similar to a system that has been previously reported [22] with a different tunable light source. The instrument is based on the Kretschmann configuration and prism coupling for generating the SPR phenomena. The wavelength-tunable light source was constructed using a tungsten-halogen lamp (HL-2000-FHSA-HP, 20 W, Ocean Optics, Dunedin, FL, USA) and a linear variable band pass filter (NT63-450 Edmund Optics, Barrington, NJ, USA) that can be tuned from 650 nm to 900 nm. The collimated incident light beam with a diameter of approximately 10 mm is p-polarized using a linear polarizer before entering the glass prism, and it illuminates the prism at a predetermined angle. The SPR sensor chip (CMD200 Xantec, Duesseldorf, Germany) was attached on the top surface of the glass prism using an index matching gel (Cargille Labs, Cedar Grove, NJ, USA). The 7-channel flow cell was made of polydimethylsiloxane (PDMS) using a precision aluminum molding technique. To prevent the adsorbed protein on the PDMS surface, the flow cell was submerged in 0.05% (w/v) Pluronic F-127 (Sigma–Aldrich, St. Louis, MO, USA) for 1 h and then rinsed with deionized water and dried with nitrogen gas. The flow cell was then applied to the top of the sensor surface via mechanical clamps. The volume of each flow cell channel was 5 μL. The buffers and samples were pumped into the flow cell by a multi-channel peristaltic pump (Ismatec, Glattbrugg, Switzerland).

The reflected images from the SPR chip were collected by a CCD camera (SCOUT scA1400-17gm Basler, Ahrensburg, Germany) at the fixed wavelength. The wavelength of the tunable light source was adjusted in a linear region of the SPR curve to obtain the highest image contrast. The selected wavelength was kept constant throughout the experiment. The relationship between the reflectivity change (*i.e.*, the SPR signal change) and the refractive index unit (RIU) was established just before the experiments were conducted using solutions with known refractive indices. Software routines based on Labview (National Instruments, Austin, TX, USA) were developed in-house for motion control and data processing.

### 2.5. Antibody Immobilization and Sample Detection

The carboxydextran surface was prepared as described by Löfås [20]. The immobilization was performed by activating the carboxydextran with 0.4 M EDC and 0.1 M NHS followed by an injection of the antibodies in sodium acetate pH 5 at a 1:10 dilution through the flow cell. The remaining available active groups were blocked by ethanolamine pH 8.5. The unbound antibodies on the surface were washed out by 5 mM NaOH followed by PBST running buffer. The bare dextran surface was used as the reference. The flow cell and the SPR sensor chip were then removed from the SPR imager. Next, the matching liquid left at the back of the sensor chip and the front surface of the prism was thoroughly cleaned. Then, the sensor chip was rotated 90° and reattached to the top of the prism. A new flow cell was then applied to the sensor chip. This way, each sample passes over all five detection strips almost simultaneously and each channel has its own reference line.

The experiments were performed by injecting 100 μL of a 5% RBC sample at a flow rate 10 μL/min. After sample injection for 12 min, during which the SPR signal reached a plateau, running buffer was passed over the surface to remove the unbound RBCs from the surface. The blood type was determined from the change of the SPR signal in the region of interest (ROI). Regeneration of the surfaces was performed using 5 mM NaOH, followed by running buffer.

## 3. Results and Discussion

### 3.1. Antibody Immobilization

We reported a simple fabrication method for a reusable polydimethylsiloxane (PDMS) multichannel flow cell that was developed for the SPR imaging machine. Multi-inlet fluidic connectors that are integrated directly onto the flow cell allow a one-step fabrication process. The connectors are based on a pressurized self-sealing PDMS mechanism, which eliminates the need for glue or epoxy. We successfully tested the PDMS flow cell by performing several SPR imaging experiments, including refractive index sensitivity and immunoassay tests.

A simple antibody array printing technique using an SPR imager and a multichannel flow cell was also demonstrated. The operating principle of the device is based on the continuous flow of the antibody samples through a PDMS multichannel flow cell that was attached directly to the top of a SPR sensor chip. All five groups of detection antibodies (see Section 2.1) can be constructed simultaneously using the procedure described above (see Section 2.5).

Five groups of detection antibodies were immobilized via covalent binding to the carboxydextran surface. Three of them were mixed clones of monoclonal antibodies, including anti-A, anti-B and anti-AB. The others were single clones of anti-A and anti-B. We predicted that the mixed-clone antibodies would provide a higher average change in the SPR signal than the single-clone antibody when a large group of samples was considered. All of the antibodies were IgM molecules that were optimized for agglutination blood-typing. All of the antibodies contained high content of bovine serum albumin (BSA); however, we found that the presence of BSA in the antibody solution did not interfere with blood-typing by the SPR imaging technique. It was found that a 1:10 dilution in acetate buffer pH 5 was the optimum concentration of the antibody for immobilization (data not shown).

### 3.2. Qualitative Analysis for Blood Typing

**Figure 1.**
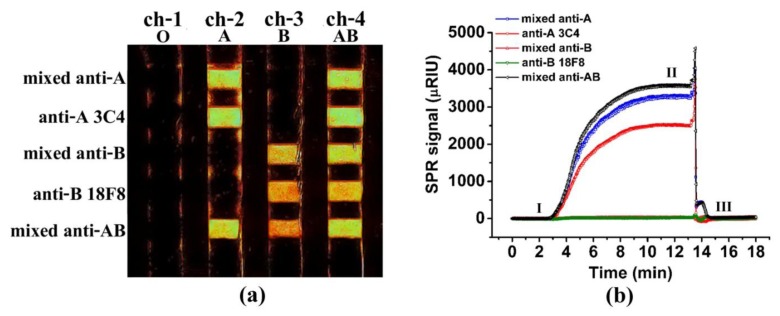
(**a**) An SPR image of the antibody array. Each row was immobilized with five different detection antibodies, and each channel represents a different RBC blood sample. A low SPR signal change is represented by a dark color. A high SPR signal change is represented by a bright color; (**b**) SPR sensorgram for the A-RBC sample (ch-2). Point I, the SPR signal at the baseline. Point II, the change in the SPR signal reached a plateau. Point III, the SPR signal returned to baseline after regeneration of the surface with 5 mM NaOH.

Agglutination-based blood-typing was performed to confirm the blood group. The results are reported in a semi-quantitative manner, *i.e.*, positive (1+, 2+, 3+ and 4+) and negative (−). All RBC samples used in the experiment were labeled either “4+” (strong agglutination) or negative (no agglutination) according to their corresponding ABO blood group. For example, A-RBCs were designated as “4+” for anti-A and negative for anti-B. RBCs bound to the immobilized antibodies were observed as bright spots on the monitor. The brighter the spot, the higher the SPR signal change, indicating that more RBCs were bound to the sensor surface (see Figure 1a). The SPR sensorgram, which is the plot of the SPR signal against time obtained from the ROIs in channel 2 (A-RBC sample), is also shown in Figure 1b.

From Figure 1a, each row from top to bottom was immobilized with the detection antibodies in the following order: mixed anti-A, anti-A 3C4, mixed anti-B, anti-B 18F8, and mixed anti-AB. Each channel from left to right was injected with the blood samples containing O-RBCs (ch-1), A-RBCs (ch-2), B-RBCs (ch-3), and AB-RBCs (ch-4). The intersecting areas of each row and channel were the defined ROIs for the bright spots for measuring the SPR signals. By observing the bright spots alone, all 60 RBC samples were typed correctly according to the ABO blood system. As seen in the figure, A-RBCs produced bright spots only for the detection line that contained anti-A, including the mixed anti-A, the anti-A 3C4, and the mixed anti-AB surfaces. Similarly, the B-RBCs produced bright spots on the mixed anti-B, the anti-B 18F8, and the mixed anti-AB surfaces. AB-RBCs gave bright spots on all five detecting lines. The O-RBCs gave no bright spots on any detection lines. Each channel has a reference line of carboxydextran. The opposite antibody to RBC can be used as a negative control. Anti-B was used as a negative control for A-RBC, anti-A was used as a negative control for B-RBC and anti-A and anti-B were used for O-RBC in each channel.

### 3.3. Quantitative Analysis

Figure 1b shows the sensorgram, a plot of the SPR signal change as a function of time, as the A-RBC sample (ch-2 in Figure 1a) passed over the detection antibodies. Point I was the baseline signal after immobilization with the detection antibodies and before the injection of the RBC sample. Point II was the point where the signal reached the plateau after injection of the A-RBC sample for 12 min. The SPR signal returned to the baseline at Point III, when the bound A-RBCs were removed from the surface using 5.0 mM NaOH and the sensor surface was ready for use in subsequent detections. We found that the sensor was suitable for reuse at least 20 times. As expected, the SPR signals increased only for the surfaces that contained anti-A (mixed anti-A, anti-A 3C4, and mixed anti-AB). There was a negligible change in the SPR signal for the surfaces without anti-A (mixed anti-B and anti-B 18F8) (Table 1).

**Figure 2.**
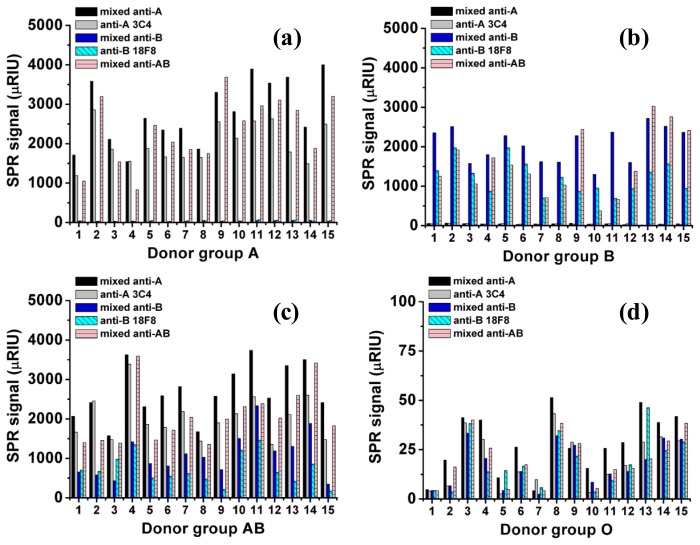
Changes in the SPR signal for all 60 samples (15 samples for each group) with all five groups of antibodies for blood types corresponding to A (**a**), B (**b**), AB (**c**) and O (**d**), respectively. Note that μRIU = 10^−6^ RIU.

The SPR signal changes for all 60 samples (15 donors for each blood group, A, B, AB, and O) are shown in Figure 2. For the O-RBC samples, the SPR signal change was very low compared with the other samples; as a result, the bar graphs are almost invisible in the figure. The signal from O-RBC to anti-A and anti-B were non-specific signals and used to define the cut off value for non-specific binding in the other blood group. Table 1 reports the averaged SPR signal changes for each blood group obtained from 15 samples (n = 15), and each sample was measured twice (in duplicate). The SPR signals showed no significant differences (paired t-test, p > 0.05, data not shown). From the table, the averaged signal changes for all the non-specific spots from O-RBC samples were 21 μRIU (21 × 10^−6^ RIU). The SPR specific signals were defined higher than the average non-specific signal of O-RBC plus three times standard deviation (3 × SD), 33 μRIU. The SPR signal changes for the corresponding specific spots were 61-85, 37-62, and 22-81 times higher than the non-specific value for the A-RBC, B-RBC, and AB-RBC samples, respectively. The signal of A-RBC samples to mixed anti-B and anti-B 18F8 surface and the signal of B-RBC samples to mixed anti-A and anti-A 3C4 surface were comparable to non-specific signal. All the non-specific signals were came from the physical adsorption of RBC to the surface which can be leached out by using a shear flow, higher flow rate. We also found that the wall shear stress higher than 1 dyne/cm^2^ (flow rate 20 μL/min) can eliminate the non specific adsorption of RBC from the sensor surface (data not shown). The SPR signal changes are in agreement with the qualitative interpretation of the agglutination results. Our results are in good agreement with those reported by Quinn *et al.* [16], where the purified antibodies rather than unpurified antibodies were used as a detection probe.

More importantly, our results showed that the use of mixed clones of antibodies as the detection probe gave a 33%–68% higher SPR signal than the use of a single clone of antibodies. These results indicated that the mixed clones of antibodies provide more binding activity and therefore, they provide a better response than a single antibody clone.

The detection principle underlying ABO blood typing by the SPR imaging technique relies on the solid-phase immobilization of the antibody probes on the sensor surface and detecting the RBCs in the solution phase by measuring the specific interaction between them. In this work, five groups of antibodies that are specific to the ABO blood group antigens were immobilized onto a carboxydextran sensor surface. The antibodies used in this work are widely implemented in the standard agglutination test. It is important to note that these antibodies contained a large portion of BSA as a result of the fetal bovine serum used during antibody culture. The standard agglutination test requires antibody titration to determine the optimum conditions for strong agglutination of the RBCs. However, in the SPR imaging technique, we found that pH optimization was needed to achieve the best antibody immobilization and reduce the amount of BSA on the surface, providing a higher SPR response. The optimal pH should be higher than the pKa of the surface and lower than the isoelectric point (pI) of the ligand [23]. We found that a 1:10 dilution of the antibody in sodium acetate buffer pH 5 was ideal for antibody immobilization. At pH 5, both BSA (pI = 4.7) and the carboxydextran surface will have a negative charge; therefore, a repulsive force occurs, preventing BSA from binding the surface. The SPR signal of RBC from the same blood group but different donors showed an unequal signal intensity towards the same antibody (Figure 2). It was believed to be due to the difference in the amount of antigens present on the RBC surface. In the AB-RBC, the different in the ratio of A antigens to B antigens in each individual play an additional role in the observed variation (Table 1). The regeneration of the sensor surface by 5.0 mM NaOH was sufficient for weak and strong binding and minimized deterioration of the antibody surface.

In addition, we also found that the effect of blood storage could be tested by comparing fresh blood samples with one-week-old and one-month-old ones. All SPR signals were comparable, with the difference between samples being less than 5%. It should be noted that the storage time has an influence on the integrity of the red blood cells, whereby the longer the storage time, the larger the amount of lysed red blood cells present in the samples. The lysed cells can be washed with normal saline and separated by centrifugation. The remaining red blood cells were used for ABO blood typing. Moreover, at a sample flow rate higher than 10 μL/min, the SPR signal change began to decrease. We reasoned that the relatively large RBC size was the main drawback. The RBCs have a biconcave shape with a longitudinal diameter of 6–8 μm and a vertical diameter of approximately 2 μm. The longitudinal diameter of the RBCs is expected to orient horizontally on the sensor surface [16]. The higher shear force exerted by the flow in the microchannel of the flow cell may explain why a high flow rate produced a decreased SPR signal compared with a low flow rate.

## 4. Conclusions

Readily available antibodies routinely used in the standard agglutination test can be used for ABO blood typing by an SPR imaging technique. The use of unpurified antibodies as a detection probe provided similar results as the use of purified antibodies described in a previous report, but the cost of the unpurified antibodies is much lower. pH optimization was required to achieve the best antibody immobilization and reduce the amount of BSA on the surface, providing a high SPR response. The use of mixed antibody clones significantly improved the SPR signal compared to the use of single clones due to the higher binding activity. In this work, all RBC samples analyzed using the SPR imaging technique were correctly grouped within approximately 12 min, and the results were consistent with those of the standard agglutination technique. The sensor surface could be regenerated more than 20 times using 5 mM NaOH, followed by running buffer. The regeneration capability of the sensor surface should further reduce the cost per test. The application of the SPR imaging technique in an array-based format has the potential for use as a high-throughput and low-cost method for ABO blood-typing. Using a sensor array technique, the blood group can be typed in a single run, and several samples can be tested in parallel.

## Figures and Tables

**Table 1.** ABO blood type results using the agglutination and SPR imaging techniques. Two groups of detection antibodies were used in the agglutination test. For the SPR imaging test, five groups of detection antibodies were used.

Samples *	**Agglutination**		SPR signal change (μRIU **)				
	
	**mixed** **anti-A**	**mixed** **anti-B**	**mixed** **anti-A**	**anti-A** **3C4**	**mixed** **anti-B**	**anti-B** **18F8**	**mixed** **anti-AB**
O-RBC	-	-	28 ± 15	20 ± 13	17 ± 11	19 ± 13	20 ± 12
A-RBC	4+	-	2789 ± 795	1998 ± 489	38 ± 9	42 ± 11	2330 ± 818
B-RBC	-	4+	41 ± 11	39 ± 12	2060 ± 425	1223 ± 405	1571 ± 770
AB-RBC	4+	4+	2687 ± 648	2026 ± 538	1078 ± 531	720 ± 371	2064 ± 680

* n = 15 for each blood group.

** μRIU = 10^-6^ refractive index units (RIU).

“-” = negative or no agglutination.
